# LinkedImm: a linked data graph database for integrating immunological data

**DOI:** 10.1186/s12859-021-04031-9

**Published:** 2021-08-25

**Authors:** Syed Ahmad Chan Bukhari, Shrikant Pawar, Jeff Mandell, Steven H. Kleinstein, Kei-Hoi Cheung

**Affiliations:** 1 Division of Computer Science, Mathematics and Science, Collins College of Professional Studies, St. John’s University, New York, NY USA; 2 Department of Genetics, Yale School of Medicine, New Haven, CT USA; 3 Program in Computational Biology and Bioinformatics, Yale School of Medicine, New Haven, CT USA; 4 Department of Pathology, Yale School of Medicine, New Haven, CT USA; 5 Department of Emergency Medicine, Yale School of Medicine, New Haven, CT USA; 6 Yale Center for Medical Informatics, Yale School of Medicine, New Haven, CT USA

**Keywords:** Ontology, Knowledgebase, Graph database, Immunology, Influenza vaccine

## Abstract

**Background:**

Many systems biology studies leverage the integration of multiple data types (across different data sources) to offer a more comprehensive view of the biological system being studied. While SQL (Structured Query Language) databases are popular in the biomedical domain, NoSQL database technologies have been used as a more relationship-based, flexible and scalable method of data integration.

**Results:**

We have created a graph database integrating data from multiple sources. In addition to using a graph-based query language (Cypher) for data retrieval, we have developed a web-based dashboard that allows users to easily browse and plot data without the need to learn Cypher. We have also implemented a visual graph query interface for users to browse graph data. Finally, we have built a prototype to allow the user to query the graph database in natural language.

**Conclusion:**

We have demonstrated the feasibility and flexibility of using a graph database for storing and querying immunological data with complex biological relationships. Querying a graph database through such relationships has the potential to discover novel relationships among heterogeneous biological data and metadata.

## Background

SQL or Structured Query Language (relational) database technology has widely been used for managing and querying data in different domains including the biomedical domain. However, NoSQL, which stands for “Not only SQL”, databases have recently emerged as an alternative database technology to address the big data problem, tackling challenges involving a large volume, velocity, variety, and veracity of data [1]. Among the NoSQL technologies, the Neo4J graph database has increasingly been used as a relationship-based (or knowledge-based), efficient, flexible and scalable method for querying and integrating data based a graph data model. Neo4J has been compared to SQL databases in terms of speed performance in querying biological data [2]. Biological data tends to be highly related, semi-structured and unpredictable, and these characteristics make the graph data model more suitable than the relational (SQL) data model. In addition, graph traversal type queries are amenable to hypothesis generation, as they can reveal relationships connecting entities that might not have been anticipated from manual examination of the network.

Using a graph representation, biological network data can be modeled naturally and manipulated efficiently. Neo4J has been utilized to represent and query network data at the molecular and cellular level. For example, Reactome [3] was converted into a Neo4J database [4] that provides a graph representation of biological pathways. Recon2Neo4j [5] is another Neo4J database that embodies the human metabolic network. It also allows translation between the Neo4J graph format and the SBML/SIF format (eXtensible Markup Language or XML format is based on the graph representation). CyNeo4j [6] extends Cystoscape [7] by using Neo4J as a more efficient network visualization and analysis engine to build a network data visualization tool.

While specific types of network data can help reveal certain aspects of a biological system [8], they can be integrated to yield a more comprehensive picture of the biological system being studied. As described in [8], hierarchical and non-hierarchical relationships exist between components in the genomic, proteomic and metabolomic network layers. Neo4J has been employed to implement a graph-based approach for relating and integrating diverse types of data for a variety of systems biology use cases. For example, Neo4J was used to integrate gene-disease and protein-drug networks to facilitate relationship-based queries to identify drug targets for asthma treatment [9]. ANIMA [10] demonstrated how to use Neo4J to create a multiscale association network from multiple data types including expression data, clinical data and biological pathways. In [8], Neo4J was used as a data warehouse to integrate a large collection of various types of genomics data from different sources.

Several projects have shown that Neo4J is an efficient graph database for storing and querying large amounts of biological network data (e.g., [2, 11, 12]). In [11], Neo4J is used to store over 700,000 Single Nucleotide Polymorphisms (SNPs). This Neo4J system takes less than one minute to execute an arbitrary count query on a dataset of 212 GB, while the best-known algorithm takes around 7 min. In another example [12], a large ontology was converted into a Neo4J graph resulting in a 13% savings on storage space and a 30-fold improvement of retrieval efficiency compared with a relational database.

There are a growing number of valuable Neo4J databases in the biological domain, but these databases are silo graphs that do not connect to facilitate integrated queries. To address this problem, we have built LinkedImm to integrate multiple types of data as a systems vaccinology use case. LinkedImm includes a Neo4J database that converts heterogeneously formatted datasets into a common graph model along with other Neo4J databases to provide an integrated view of the data.

## Results

**Fig. 1:**
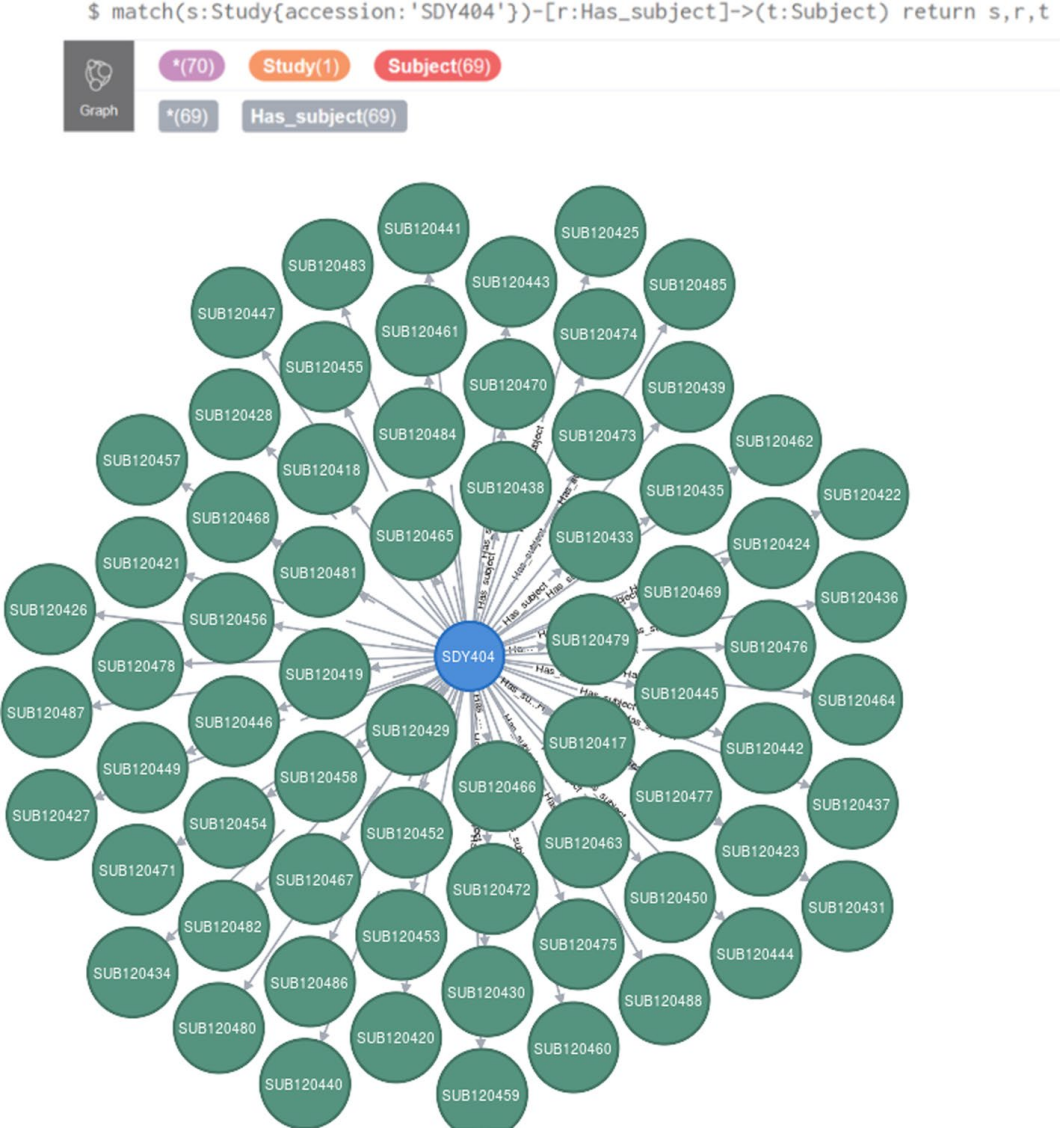
A Cypher query (top line) is demonstrated to identify all the subjects (red circles) profiled in a specified study (SDY404, orange circle)

**Fig. 2:**
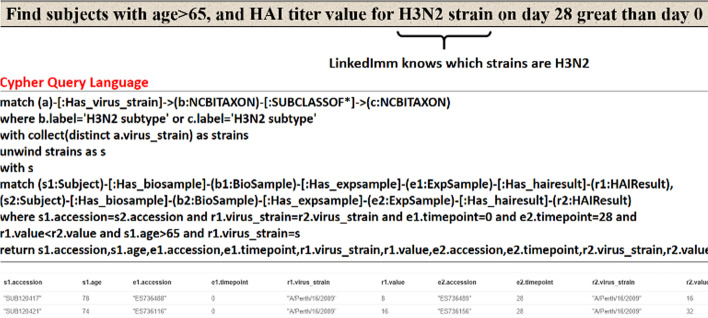
A more expressive Cypher query is used to make use of inference based on the hierarchical relationship recognizing virus strains of the H3N2 subtype

The LinkedImm graph currently has 37,992 nodes and 72,229 relationships. A graph (network) of this size benefits from the use of Neo4J as a graph database engine for efficiency of graph data querying and manipulation. Neo4J also comes with a web-based interface for users to write Cypher queries against the graph database. The query output can be displayed as a graph or in other formats (e.g., tabular format). Figure 1 shows a Cypher query to return the subjects for a specific study whose accession is “SDY404” and its output as a graph. The Cypher query language is expressive enough to let users pose more sophisticated queries like the following: “find subjects with age greater than 65 years old and HAI titer values for H3N2 virus strains on day 28 greater than day 0” (Fig. 2). Not only does this query requires connecting and filtering multiple types of information (e.g., demographic information and HAI titer values measured at different time points), but it also makes inference based on the taxonomic hierarchy of virus strains. In this case H3N2 is a broader subtype of different virus strains like A/Victoria/3/1975 and A/Perth/16/2009. The system goes beyond virus strain name matching only, as it automatically retrieves all the virus strains which fall under the H3N2 subtype.

**Fig. 3:**
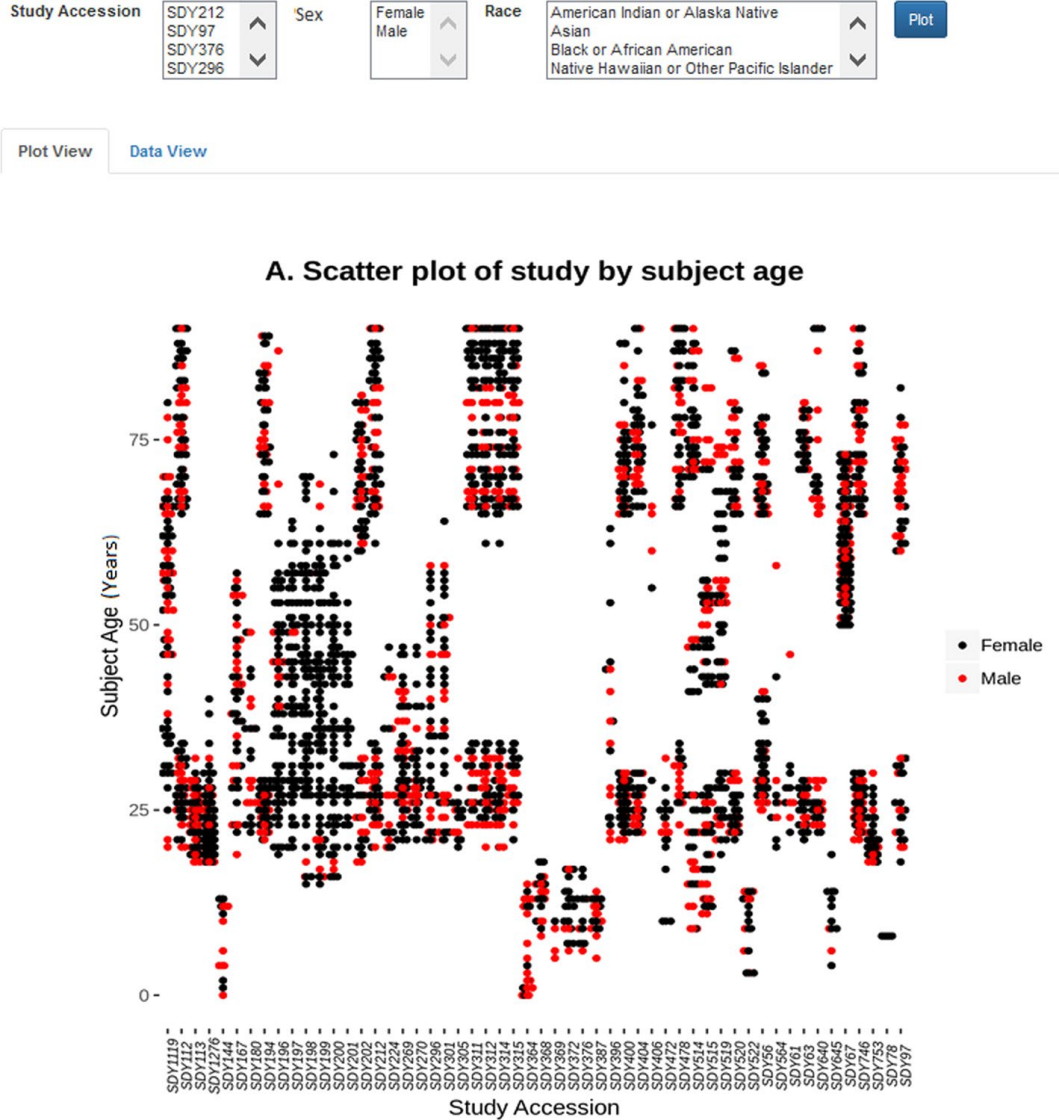
A plot view of subjects’ age distributions for different influenza vaccination studies

**Fig. 4:**
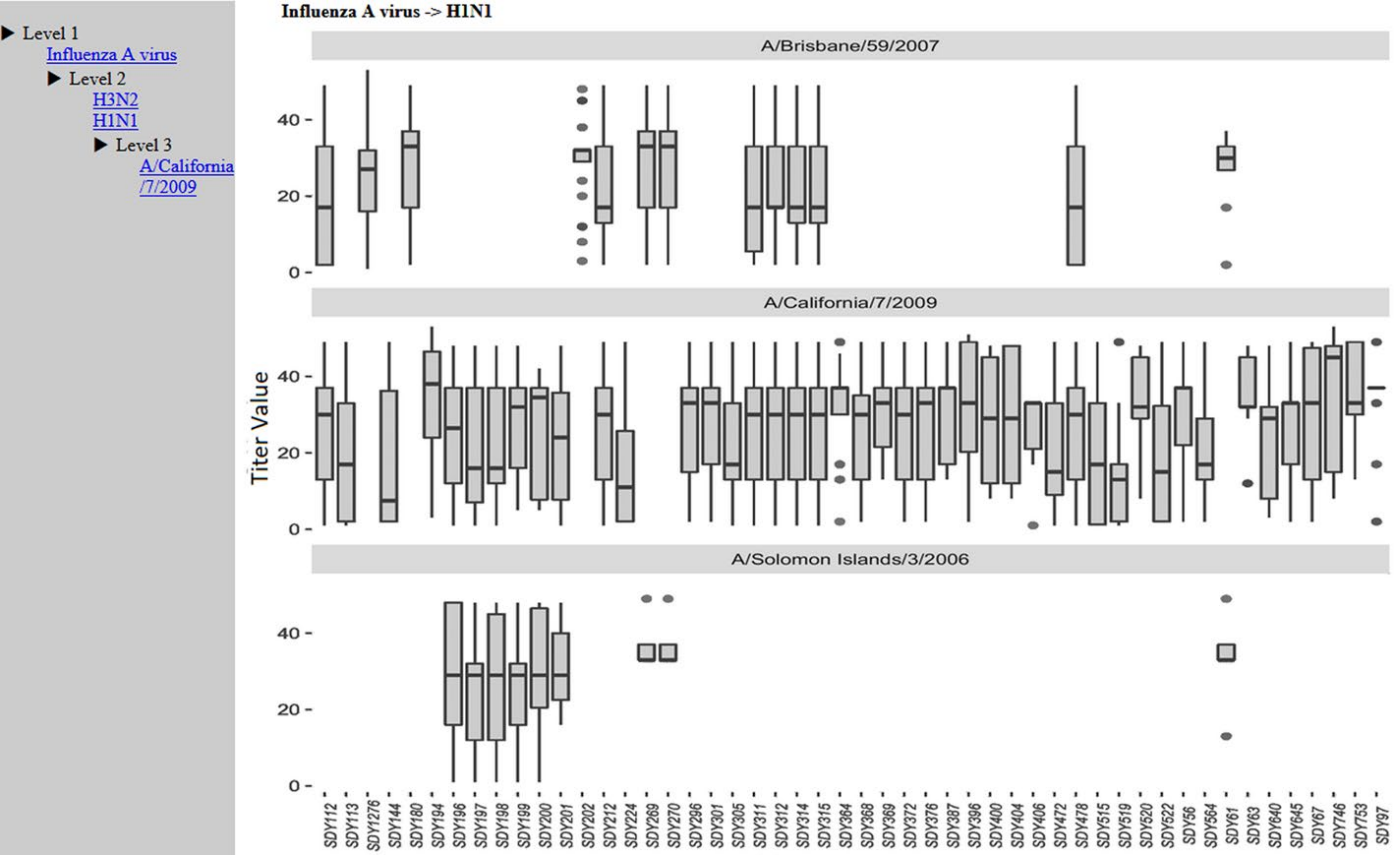
A box plot of the distributions of antibody titer values for different H1N1 virus strains (*A/Brisbane/59/2007, A/California/7/2009,* and *A/Solomon Islands/3/2006*) measured across different studies

Although the Cypher query language is expressive, it requires a learning curve which can be high for general users, like experimental immunologists, who may not be familiar with any database query language. To remove this barrier, we have developed a web-based dashboard for users to interact with LinkedImm in a more intuitive way. The design of this dashboard is to provide basic subject-level information, including age distributions (Fig. 3) and antibody titers (Fig. 4) across the different studies. The user can perform data filtering to identify specific studies and/or human subjects. The antibody titer interface shows the capability of linking the immune profiling measurements with external, prior knowledge on virus strains. The virus strains included in the influenza vaccine can change each year, but these strains can be related to each other based on their type (e.g., influenza A or B) and subtype (e.g., H1N1 or H3N2). With LinkedImm, users can query data on individual virus strains, but also data from different types and subtypes of virus strains including the H1N1 strains (Fig. 4).

**Fig. 5:**
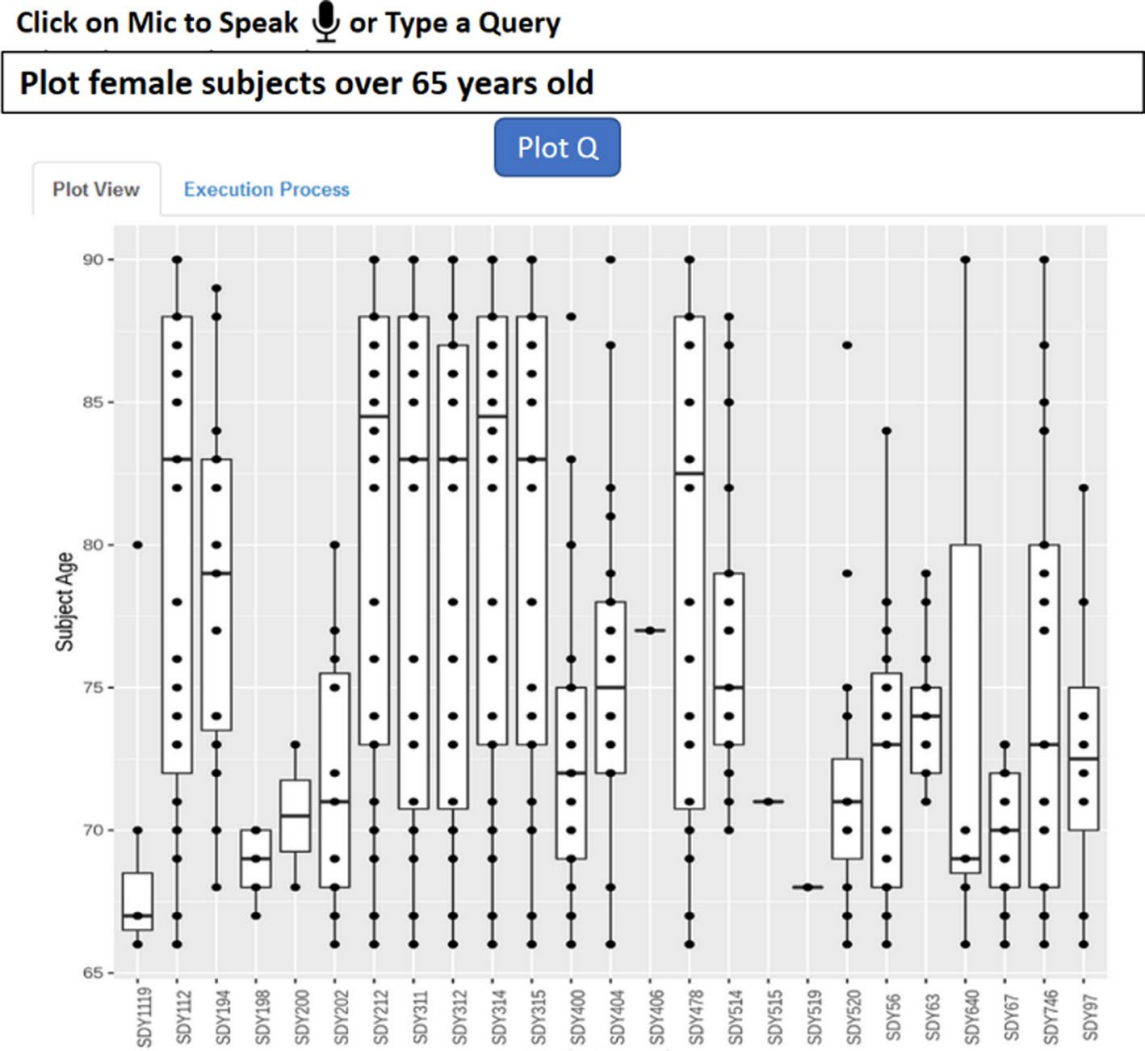
A natural language query example illustrating a plot female subjects over 65 years old

Our prototype web interface allows the user to pose ad-hoc database queries either through a form or through spoken/typed natural language. Figure 3 demonstrates the use of a form-based query to visualize the distribution of subject ages in a collection of studies. The “Data View” tab displays data in tabular format, which can be downloaded as a CSV file by clicking the download link. Figure 5 shows a natural language query resulting in a plot of the age distribution of female subjects whose age is older than 65.

**Fig. 6:**
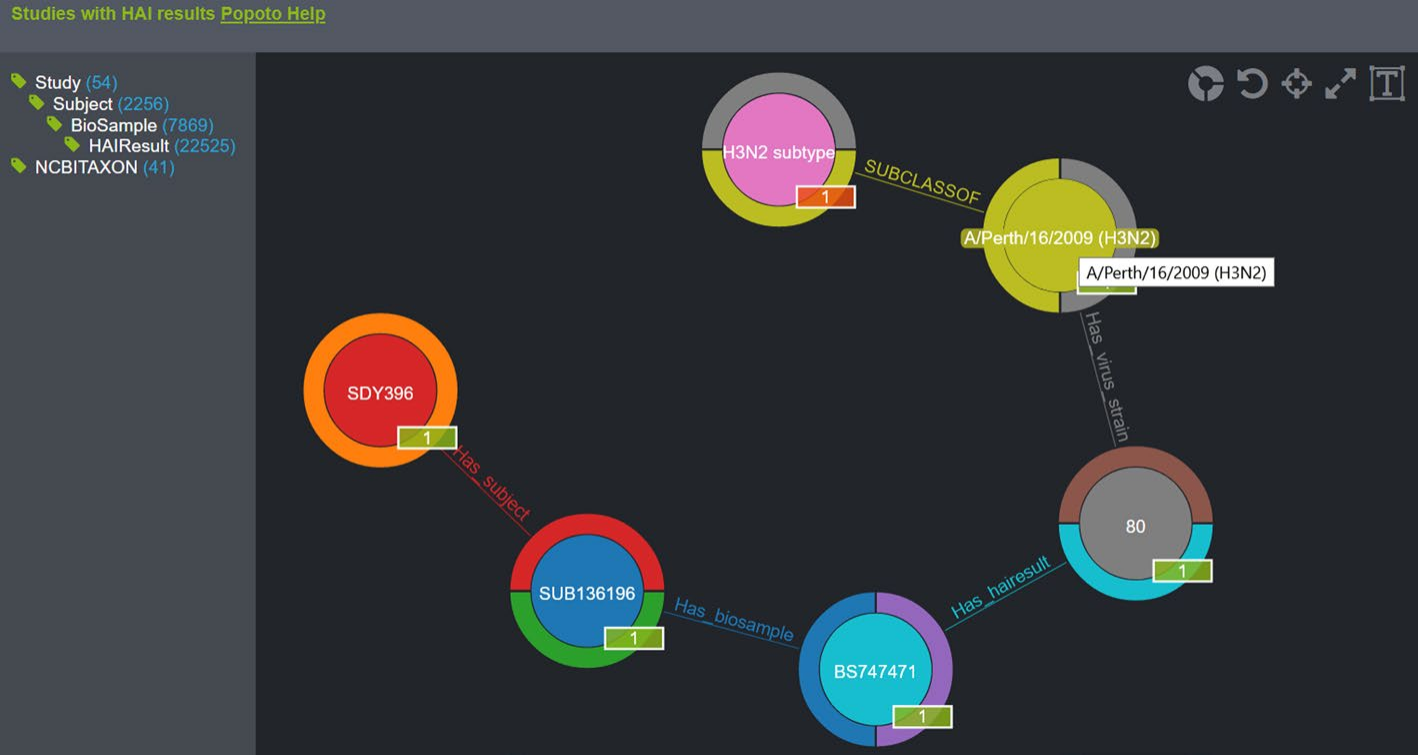
Query visualization of the LinkedImm graph

As LinkedImm is a graph database, it is natural to view the graph structure and query based on the underlying graph structure. To this end, we have implemented a graph query visualization interface using popoto.js [13], which is a JavaScript-based method for dynamic visualization and queries of graph data. Figure 6 shows an example of a graph query including a path for a given study (SDY396), a subject’s HAI measurement value of the virus strain “A/Perth/16/2009” which is a subclass of the H3N2 subtype as defined in the NCBI TAXON. The left panel shows the graph structure in a hierarchical manner.

## Discussion

LinkedImm is a graph-based framework for building a web-accessible knowledgebase that integrates related types of immunological data from multiple resources including other unconnected Neo4J databases. This integrated resource can be accessed in multiple ways through the web using Cypher queries, a dashboard interface, and natural language query interface.

While data and metadata can be represented directly as a graph, further enrichment can be achieved by introducing additional components to the graph based on existing concepts and relationships. For example, we have added a graph component to define older subjects as subjects whose age is greater than 65 years old. Similarly, we can add new gene nodes to classify genes as “up-regulated genes” if their expression values meet some criteria post-vaccination that we specify in advance, such as being at least two-fold higher compared with the pre-vaccination sample. These enriched components (subgraphs) can be generated using Cypher queries so that the resulting enriched graph can further be queried in a more straightforward manner.

An intuitive user interface is needed in order to make graph databases widely accessible to scientists and researchers (e.g., experimental immunologists). To this end, we have implemented a web-based dashboard, visual graph query interface, and a natural language query interface as an intuitive approach to easing data navigation and queries. More advanced data visualization methods can be explored to interrogate and view graph/network data.

The current size of LinkedImm does not require extensive computing power. Most queries (including the one involving virus strain inferencing shown in Fig. 2) can be executed within several seconds. As LinkedImm continues to expand, speed performance may become critical. To boost the hardware speed, we can increase the main memory size, upgrade CPU speed, and utilize solid state disks. In addition, Neo4J comes with an enterprise version that provides causal clustering [14] with fault tolerance, scalability, and data consistency.

As more Neo4J databases/knowledgebases are publicly available in various domains, the need to integrate these graph databases increases. It would be desirable to create a central registry or repository that can help users find these graph databases and a common query interface to access multiple graph databases simultaneously. Linking the entities in these databases can be a challenge. In the current LinkedImm system, we semi-automatically match the names between entities with some manual intervention. However, a more intelligent and automatic approach could semantically map study data to ontologies, thus unifying terminologies and standardizing relationships to facilitate data integration. Neosemantics [15] is a Neo4J plugin that has been developed to map Neo4J databases to RDF/OWL ontologies. This would allow a seamless integration between Neo4J and RDF graphs including those in the linked data cloud [16]. While the main focus of LinkedImm is data integration, there is a possibility of integrating graph databases with analytic services. For example, the Neo4J BI Connector [17] allows direct access to Neo4J graph data from analytic tools such as Tableau [18]. We can potentially integrate machine learning tools to perform graph data mining (e.g., [19]).

## Conclusions

We have used Neo4J to build LinkedImm, a graph knowledgebase system to facilitate systems vaccinology research by integrating diverse types of immunological data from multiple sources. LinkedImm is publicly accessible at [20]. We have demonstrated how a graph database can be used to link data of different types and make inferences based on meaningful relationships. While SQL databases are an industry standard and have been used widely, NoSQL databases like Neo4J have gained increasing attention, traction and momentum because of their advantages in dealing with diverse, semi-structured data. In addition to database technologies, we have shown various ways a user can interact with the LinkedImm system, including a web-based dashboard interface, graph query visualization, and natural language query interface.

## Methods

Our methods mainly involve two components: (i) the creation of a graph database by converting and integrating diverse types of immunological data in heterogeneous formats from multiple sources into a common graph model, and (ii) the use of web-based and voice-based technologies to build an intuitive interface for users to interact, query, and visualize the graph database.

### Systems vaccinology use case

**Fig. 7:**
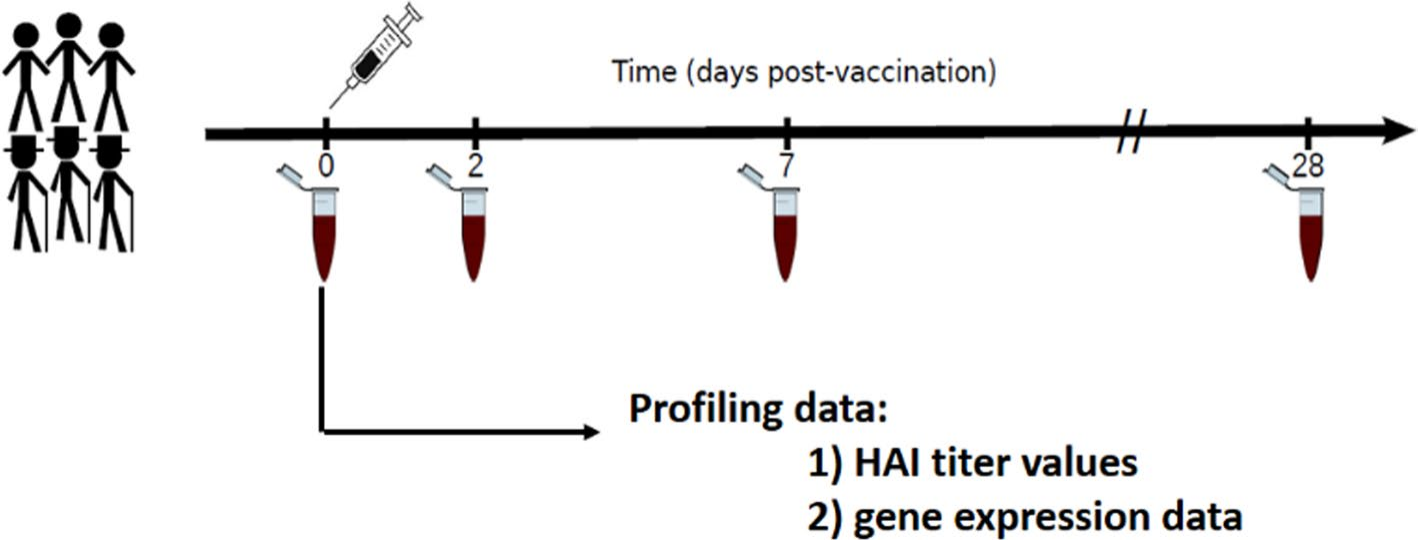
Generation and collection of profiling data for a time-series influenza vaccination cohort study consisting of young and older subjects who are represented by the different stick figures

Systems vaccinology studies make use of high-throughput profiling methods to provide an integrated, dynamic view of the immune response to vaccination [21]. Systems vaccinology studies typically involve collection of blood samples from well characterized cohorts at multiple time-points pre- and post-vaccination (Fig. 7). Systems-level immune profiling techniques, such as genome-wide transcriptional profiling or B/T cell receptor repertoire profiling [21], are carried out on each of the samples in order to measure the resulting immune response. These data are then analyzed to identify features that are altered as a result of vaccination or are associated with the quality of the induced immune response.

Systems vaccinology approaches have been used by many groups to understand the human response to influenza vaccination [21]. Influenza, a highly contagious respiratory disease caused by influenza viruses, is a global health concern. There are 30,000 to 40,000 annual deaths caused by influenza in the United States [22]. The primary method of preventing influenza in vaccination. However, despite the clear public health success of annual influenza vaccinations, a high fraction of individuals, particularly older individuals, fail to induce a significant antibody response [23, 24].

The NIH/NIAID Human Immunology Project Consortium (HIPC) has carried out coordinated several influenza vaccination profiling studies [25]. HIPC has also performed a meta-analysis that identified a pre-vaccination transcriptional signature that predicted the quality of the response as measured by antibody titers [25]. The data associated with these studies are stored in the NIH/NIAID ImmPort repository [26]. ImmPort is a MySQL relational database. HIPC data are also made available to the public through ImmuneSpace. ImmuneSpace pulls data from ImmPort and provides an interface that facilitates user-driven analyses of the data [25]. ImmPort and ImmuneSpace provide a wealth of data for secondary analysis that could be used to identify signatures of vaccination responses, including samples from pre- and post-vaccination time-points.

A search of ImmPort identified over fifty influenza vaccination studies with pre- and post-vaccination transcriptional profiling data. These include both HIPC and non-HIPC studies. We have initially focused on this set of influenza vaccination studies to construct LinkedImm. These studies followed the typical systems vaccinology design (Fig. 7). The studies focused on young adult cohorts, although some also included older adults. Demographic data (e.g., age, race and sex) are available for most subjects, along with the measured antibody titers and transcriptional profiling data. Titers of antibodies for each of the influenza strains included in the vaccine are most often measured by hemagglutination-inhibition assay (HAI) at one time-point pre-vaccination (typically day 0) and a second time-point post-vaccination (typically day 28). This allows for assessing the quality of the vaccination response. Many of these studies also carry out transcriptional profiling on PBMCs using gene expression microarrays at several time-points pre- and post-vaccination (time point 0 represents pre-vaccination and subsequent time points correspond to the post-vaccination period). Each of these experiments yields expression level measurements for each of ~ 20,000 genes. Additional types of immune profiling data are also available for many of these studies, such as high-dimensional cytometry, but these have been excluded from the prototype LinkedImm system. Other studies in ImmPort use different experimental technologies, such as virus neutralization assay (VNA) and RNA-seq, make similar antibody titer and gene expression measurements, respectively.

**Fig. 8:**
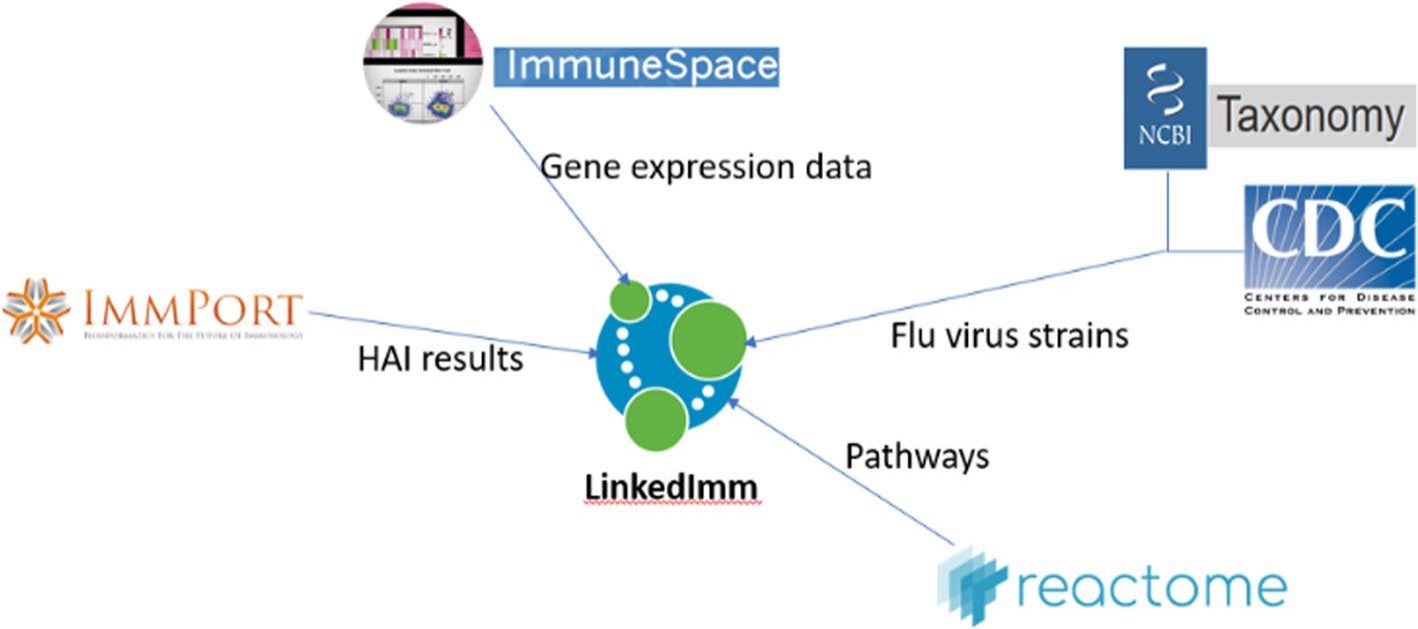
The LinkedImm system features integration of diverse types of data from multiple sources

**Fig. 9:**
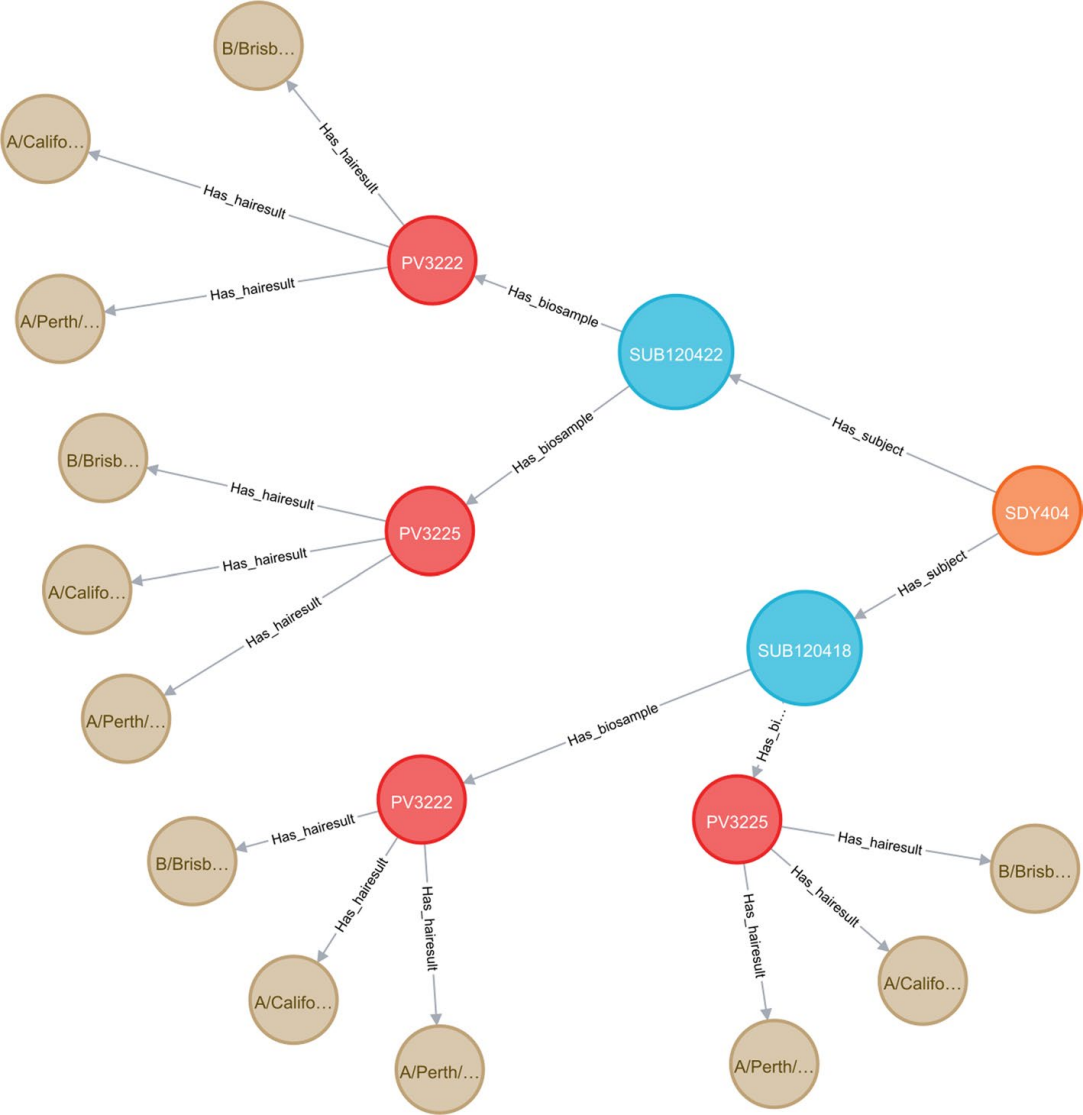
The graph model of HAI measurements for a single study (SDY404). Study subjects (red circles) are associated with their demographic information, including age cohort (e.g., young, older, etc.). Each subject is linked to blood samples that were collected (blue circles). HAI measurements (brown circles) are collected for each of the virus strains included in the vaccine given to that subject

Figure 8 shows the types of data that are currently integrated into the LinkedImm system The construction of LinkedImm begins by converting ImmPort’s HAI and associated gene expression data into a Neo4J graph database. The conversion steps and the resulting graph model are shown in Fig. 9. In our graph model, Study is represented as the root node that has Subject as its child node (subjects can belong to a cohort or arm). Subjects are linked to biosamples (Biosample) that have experimental samples (Expsample) with which assay results are associated. The current implementation of LinkedImm features the Enterprise Edition (version 3.5) of Neo4J running on a Linux server with 32 GB RAM, and 500 TB Solid State Disk (SSD). In addition, it uses Apache 2.0 as the web server. We programmatically extracted HAI data from ImmPort using its API (application program interface) that allows us to retrieve HAI results from all vaccine studies (there are a total of 58 studies with HAI results). The API returns query output in CSV format. We wrote a Cypher script to convert the CSV file that contains the HAI results into the Neo4J graph format. Unlike the case for HAI data, ImmPort only stores metadata about the microarray experiment. The underlying gene expression data are stored in the NCBI Gene Expression Omnibus (GEO) repository [27]. Links have been established at the experiment sample level between ImmPort and GEO. To obtain the gene expression data, we leverage ImmuneSpace [28], which uses the metadata of published studies from ImmPort to retrieve the raw data from GEO and then processes these data to produce normalized gene expression values. Further analysis (e.g., fold change) can be performed on the processed gene expression data obtained from ImmuneSpace. In this work, the graph database only stores the decisions for up/down regulation based on the fold change data computed from the processed data obtained from ImmuneSpace. The result of this process is a graph database that links subjects, biosamples and experiment samples, along with gene expression and HAI results (see Fig. 9).

Integrative analysis is a key enabler for systems vaccinology. For example, identifying pathways that are activated following vaccination requires combining information on gene expression levels with prior knowledge on the set of genes associated with each pathway. Many pathway databases (e.g., Reactome [29]) that collect prior knowledge have been developed and made publicly available over the web for researchers to use. While web-accessible databases can be individually queried and the query results can be downloaded in different formats, it is often the user’s responsibility to manually or programmatically link them in a meaningful context for integrated data analysis. Such data integration efforts can be labor-intensive and hindered by the lack of standard formats and identifiers, as well as the lack of formal data relationships between different databases.

**Fig. 10:**
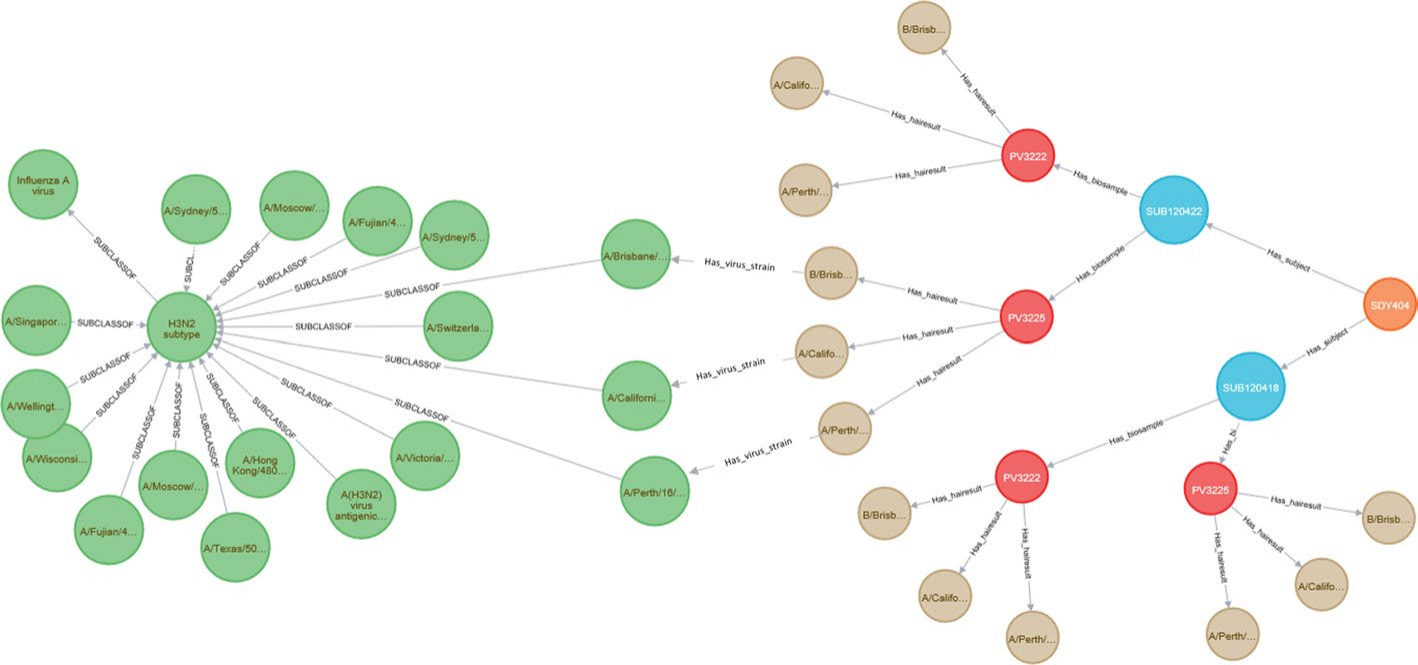
The graph model for HAI result linked to NCBI TAXON’s virus taxonomy

To facilitate integrative analyses, we have expanded the LinkedImm graph database by linking the HAI and gene expression data from ImmPort with other types of data available through different sources (Fig. 8). Specifically, we have done the following: (1) define relationships between HAI measurements in different studies by including additional information on influenza virus strains obtained from the CDC and NCBI, and (2) incorporate pathway associations from Reactome to define the relationships between genes by [3]. The seasonal influenza virus strain information was produced by WHO and made available as a tab-delimited file by the CDC. We read in this file, and automatically matched the virus strain names to the names of the virus strains in the Neo4J graph for NCBI Taxonomy provided by EBI [30] (Fig. 10). For efficiency, we extracted a subgraph of NCBI Taxonomy based on the WHO virus strains, which is stored in LinkedImm. Second, we imported a subgraph of the Reactome graph database into LinkedImm. This subgraph includes every pathway that contains at least one gene measured in the gene expression data from ImmPort. In total, the LinkedImm graph database encompassed information on nineteen unique influenza virus strains and over two thousand human pathways covering approximately twenty thousand genes.

### Web-Based dashboard and natural language query interface

To allow users to explore LinkedImm without using the Cypher query language, we have used the PHP framework to develop intuitive web interfaces. These interfaces provide web forms for users to specify the information they want to retrieve and display. The user’s specification is passed to the dynamic Cypher generation (DCG) function which subsequently queries the LinkedImm knowledge graph and returns the query results in different formats (including CSV files and graphical plots). The CSV files are converted into graphical plots using R and Ggplot2 library.

LinkedImm also provides the ability to issue queries in a natural language. Through our natural language interface, the user can enter a natural-language query in a text input field. Specifically, we have used the Google Chrome Web Speech API to implement this speech-to-text conversion. In this case, by using the Chrome web browser, the user can speak through their microphone to pose natural language queries.

We have used Dialogflow [31] (a service owned by Google) as a natural language processing (NLP) agent to translate a natural-language query into the corresponding Cypher query. We have trained the Dialogflow agent with several possible query combinations which users potentially could ask to access the information using the LinkedImm natural language query interface. The parsed output from the Dialogflow is fed back to DCG which then generates the Cypher query to retrieve the data from the Neo4J database. The query resultsare displayed as interactive plots and tables. The NLP agent has some ability to remember the current query context. Hence, it could be further trained to implement support for follow-up queries. For example, “How about for 2014?” after having previously asked the agent to show results from the 2013 influenza vaccination season.

## Data Availability

LinkedImm is publicly accessible at http://linkedimm.org. All the data and source code for implementing HAI analysis, natural language query, visual graph query and demographics dashboard can be obtained at bitbucket here: https://bitbucket.org/kleinstein/LinkedImm

## Abbreviations

ANIMA: Association Network Integration for Multiscale Analysis
API: Application Programming Interface
CDC: Centers for Disease Control and Prevention
CPU: Central Processing Unit
CSV: Comma Separated Values
DCG: Dynamic Cypher generation
EBI: European Bioinformatics Institute
GEO: Gene Expression Omnibus
HAI: Hemagglutination Inhibition Assay
HIPC: Human Immunology Project Consortium
ImmPort: Immunology Database and Analysis Portal
NCBI TAXON: National Center for Biotechnology Information Taxonomy
NIH: The National Institutes of Health
NLP: Natural Language Processing
NIAID: The National Institute of Allergy and Infectious Diseases
NoSQL: Not only SQL
OWL: Web Ontology Language
PBMC: Peripheral Blood Mononuclear Cell
PHP: Hypertext Preprocessor
RAM: Random Access Memory
RDF: Resource Description Framework
SBML: Systems Biology Markup Language
SIF: Simple Interaction File
SQL: Structured Query Language
WHO: World Health Organization
